# Explainable Machine Learning to Predict Successful Weaning Among Patients Requiring Prolonged Mechanical Ventilation: A Retrospective Cohort Study in Central Taiwan

**DOI:** 10.3389/fmed.2021.663739

**Published:** 2021-04-23

**Authors:** Ming-Yen Lin, Chi-Chun Li, Pin-Hsiu Lin, Jiun-Long Wang, Ming-Cheng Chan, Chieh-Liang Wu, Wen-Cheng Chao

**Affiliations:** ^1^Department of Information Engineering and Computer Science, Feng Chia University, Taichung, Taiwan; ^2^Division of Chest Medicine, Department of Internal Medicine, Taichung Veterans General Hospital, Taichung, Taiwan; ^3^Department of Life Sciences, National Chung-Hsing University, Taichung, Taiwan; ^4^Division of Critical Care and Respiratory Therapy, Department of Internal Medicine, Taichung Veterans General Hospital, Taichung, Taiwan; ^5^Central Taiwan University of Science and Technology, Taichung, Taiwan; ^6^The College of Science, Tunghai University, Taichung, Taiwan; ^7^Department of Critical Care Medicine, Taichung Veterans General Hospital, Taichung, Taiwan; ^8^Department of Computer Science, Tunghai University, Taichung, Taiwan; ^9^Department of Automatic Control Engineering, Feng Chia University, Taichung, Taiwan

**Keywords:** explainable AI, weaning, prediction mode, prolonged mechanical ventilation, machine learning

## Abstract

**Objective:** The number of patients requiring prolonged mechanical ventilation (PMV) is increasing worldwide, but the weaning outcome prediction model in these patients is still lacking. We hence aimed to develop an explainable machine learning (ML) model to predict successful weaning in patients requiring PMV using a real-world dataset.

**Methods:** This retrospective study used the electronic medical records of patients admitted to a 12-bed respiratory care center in central Taiwan between 2013 and 2018. We used three ML models, namely, extreme gradient boosting (XGBoost), random forest (RF), and logistic regression (LR), to establish the prediction model. We further illustrated the feature importance categorized by clinical domains and provided visualized interpretation by using SHapley Additive exPlanations (SHAP) as well as local interpretable model-agnostic explanations (LIME).

**Results:** The dataset contained data of 963 patients requiring PMV, and 56.0% (539/963) of them were successfully weaned from mechanical ventilation. The XGBoost model (area under the curve [AUC]: 0.908; 95% confidence interval [CI] 0.864–0.943) and RF model (AUC: 0.888; 95% CI 0.844–0.934) outperformed the LR model (AUC: 0.762; 95% CI 0.687–0.830) in predicting successful weaning in patients requiring PMV. To give the physician an intuitive understanding of the model, we stratified the feature importance by clinical domains. The cumulative feature importance in the ventilation domain, fluid domain, physiology domain, and laboratory data domain was 0.310, 0.201, 0.265, and 0.182, respectively. We further used the SHAP plot and partial dependence plot to illustrate associations between features and the weaning outcome at the feature level. Moreover, we used LIME plots to illustrate the prediction model at the individual level. Additionally, we addressed the weekly performance of the three ML models and found that the accuracy of XGBoost/RF was ~0.7 between weeks 4 and week 7 and slightly declined to 0.6 on weeks 8 and 9.

**Conclusion:** We used an ML approach, mainly XGBoost, SHAP plot, and LIME plot to establish an explainable weaning prediction ML model in patients requiring PMV. We believe these approaches should largely mitigate the concern of the black-box issue of artificial intelligence, and future studies are warranted for the landing of the proposed model.

## Background

Mechanical ventilation (MV) is one of the essential organ support management approaches in critically ill patients, and ~5–10% of patients receiving MV require prolonged MV (PMV), defined as using MV for more than 21 days (1, 2). There is an increasing health burden of PMV globally, and the estimated economic burden in the United States was nearly 25 billion per year (3–5). It has been estimated that merely 50% (95% confidence interval [CI] 47–53%) of patients with PMV can be liberated from MV (6); however, the study to predict weaning outcome in patients under PMV remains scarce despite of an increasing health impact of PMV.

Artificial intelligence (AI) is widely applied in various fields, but the black-box issue remains the main concern for the application of AI in the medical field (7, 8). Recently, explainable AI algorithms, including our recently published research in critically ill influenza patients, have been increasingly applied to interpret the AI model based on *post-hoc* analyses and domain knowledge, and the black-box issue can largely be mitigated (9, 10). Due to the steadily increasing number of patients requiring PMV in Taiwan during the last two decades, a specialized unit, respiratory care center (RCC), has been established to facilitate weaning in patients with PMV (4, 11). In the present study, we aimed to use electronic medical records of an RCC in central Taiwan collected between 2013 and 2018 and an explainable machine learning (ML) approach to establish a weaning prediction model in patients requiring PMV.

## Methods

### Ethical Approval

This study was approved by the Institutional Review Board of the Taichung Veterans General Hospital (TCVGH: CE19072A). All data were obtained from electronic medical records and anonymized before analyses, and informed consent was hence waived.

### Study Population

This retrospective study was conducted at TCVGH, a tertiary-care referral hospital with ~1,500 beds, six intensive care units (ICUs), and one 12-bed RCC in central Taiwan. All patients who had been admitted to the study RCC for a first attempt at weaning between 2013 and 2018 were enrolled in the study. Liberation from MV for five consecutive days was defined as successful weaning given that one Taiwanese population-based study has shown high durability of weaning success after liberation from the ventilator for 5 days in patients with PMV (12).

### Variables Categorized by Main Clinical Domains

The dataset was established through collecting electronic medical records during the first index admission to RCC, and the first day with MV was defined as day 1 of the index admission. Data were censored after the patient was discharged from RCC, including successful weaning, mortality, or being transferred back to the ICU/ward in ventilator-dependent status. The dataset mainly consisted of five clinical domains: (1) ventilation domain (weekly average fraction of inspired oxygen [FiO_2_, %], positive end-expiratory pressure [PEEP, cmH_2_O], peak inspiratory pressure [Ppeak, cmH_2_O], mean airway pressure [Pmean], tidal volume per predicted body weight [VT/PBW, ml/kg], respiratory rate, and minute ventilation); (2) fluid domain (weekly fluid balance data, including input, feeding amount, urine output, hemodialysis output, and overall fluid balance); (3) physiology domain (weekly average blood pressure, heart rate, body temperature, oxygen saturation [SaO_2_], and glucose levels); (4) lab domain (main laboratory data, including albumin, white blood cell counts, hemoglobin concentration, platelet counts, liver function tests, and renal function tests); and (5) others, including Acute Physiology and Chronic Health Evaluation (APACHE) II score, comorbidities, and medications.

### Extreme Gradient Boosting (XGBoost)

We used XGBoost to construct a weaning outcome prediction model. Gradient boosting methods including XGBoost employed iterative combinations of ensembles of weak prediction models into one strong learner (13). XGBoost further applies a second-order Taylor series to approximate the value of the loss function and reduces the potential overfitting by application of regularization (14). In the setting of the hyperparameters, the optimal values were identified by a grid search on potential value combinations of the parameters. The key fine-tuned parameters in the present study included the number of trees (n_estimator = 770), learning rate (eta = 0.01), and maximum tree depth (max_depth = 3) (see Supplementary Table 1 for detailed parameters) (14).

### Random Forest (RF)

In addition to XGBoost, we also employed another tree-based classifier, namely, RF. These two ML models have crucial differences in the ensemble method. In brief, XGBoost is based on the ensemble of weak learners, whereas RF is based on fully grown decision trees (13, 15). In RF, n_estimator was 100, max_depth was 4, and default values were applied for the other parameters in RF as well as logistic regression (LR) (see Supplementary Table 1 for detailed parameters in RF).

### LR

LR is a widely used statistical method in medicine and is frequently used as an ML model for classification tasks. LR mainly based on the assumption that a linear relationship exists between the input variables and the outcomes (16). (see Supplementary Table 1 for detailed parameters in LR).

### SHapley Additive Explanations (SHAP)

To illustrate the strength and direction of associations between features and the weaning outcome, we implemented SHAP, which is an increasingly used *post-hoc* approach to explain the output of the ML model (17). In brief, SHAP is an additive feature attribution method that gives an explanation of the tree ensemble's overall impact in the format of the contribution of a feature, and the visualized presentation of the SHAP plot is relatively in line with human intuition. Moreover, we also used the partial dependence plot (PDP) to show the marginal effect of features on the predicted outcome.

### Local Interpretable Model-Agnostic Explanations (LIME)

We also used LIME to illustrate the impact of key features at the individual level (18). In brief, LIME provides an explanation of a classifier through approximating the key features by applying a local linear model. The output of LIME is a list of explanations that indicate the contribution of key features to the predicted outcome in an individual patient.

### Statistical Analysis

Categorical data were expressed as frequencies (percentages), and continuous data were presented as means ± standard deviations. Differences between successful weaning and failed weaning were analyzed using Student's *t*-test for continuous variables and Fisher's exact test for categorical variables. Data of 80% of randomly selected patients were used as the training dataset, and the testing set consisted of data of the remaining 20% of the patients (see Supplementary Figure 1 for the flow diagram of the study). The performance of ML models to predict weaning outcome was determined by using the area under the receiver operating characteristic (ROC) curve (AUC). For the interpretability of the ML models, feature importance was quantified and categorized by clinical domains. In the present study, the score of feature importance was determined by the average gain across all splits of a feature used in the construction of the tree-based model. Furthermore, we used the SHAP summary plot and partial SHAP dependency plot for a visualized interpretation of each feature. We also employed LIME plots for visualized interpretations at the individual level. Python version 3.6 was used in the present study.

## Results

### Demographic and Ventilatory Data

A total of 963 patients requiring PMV were enrolled, and 300 features were used in the present study. The mean age of enrolled patients was 69.3 ± 16.0 years, and 64.2% (618/963) of patients was female. We found that 56.0% (539/963) of patients requiring PMV were weaned from MV. Patients with unsuccessful weaning were more likely to have congestive heart failure (17.5 vs. 11.1%, *p* < 0.01), atrial fibrillation (22.4 vs. 14.5%, *p* < 0.01), chronic obstructive pulmonary disease (19.1 vs. 11.1%, *p* < 0.01), end-stage renal disease (13.7 vs. 8.2%, *p* < 0.01), active malignancy (23.6 vs. 14.7%, *p* < 0.01), and a higher APACHE II score on RCC admission (19.4 ± 5.7 vs. 16.5 ± 5.1, *p* < 0.01) compared with those who were successfully weaned from MV (Table 1). Table 2 summarizes weekly average ventilatory parameters between weeks 4 and 9 at the RCC in patients with PMV. Patients successfully weaned from MV tended to have a lower FiO_2_, PEEP, Ppeak, Pmean, VT/PBW, and minute ventilation than those who remained ventilator dependent, whereas the respiratory rate was similar between the two groups (Table 2).

**Table 1:** Characteristics of the 963 patients categorized by weaning outcome.

	**All**	**Successful weaning (–)**	**Successful weaning (+)**	***p*-value**
	***N* = 963**	***N* = 424**	***N* = 539**	
**Demographic data**				
Age (years)	69.3 ± 16.0	72.1 ± 14.3	67.1 ± 16.8	<0.01
Sex (female)	618 (64.2%)	291 (68.6%)	327 (60.7%)	0.01
Body mass index	22.5 ± 4.5	22.6 ± 4.6	22.4 ± 4.5	0.52
**Comorbidities**				
Hypertension	538 (55.9%)	236 (55.7%)	302 (56.0%)	0.91
Diabetes mellitus	329 (34.2%)	152 (35.8%)	177 (32.8%)	0.33
Congestive heart failure	134 (13.9%)	74 (17.5%)	60 (11.1%)	<0.01
Atrial fibrillation	173 (18.0%)	95 (22.4%)	78 (14.5%)	<0.01
COPD	141 (14.6%)	81 (19.1%)	60 (11.1%)	<0.01
Asthma	38 (3.9%)	17 (4.0%)	21 (3.9%)	0.93
End-stage renal disease	102 (10.6%)	58 (13.7%)	44 (8.2%)	<0.01
Liver cirrhosis	29 (3.0%)	12 (2.8%)	17 (3.2%)	0.77
Cerebral vascular disease	254 (26.4%)	122 (28.8%)	132 (24.5%)	0.13
Malignancy (inactive)	77 (8.0%)	31 (7.3%)	46 (8.5%)	0.49
Malignancy (active)	179 (18.6%)	100 (23.6%)	79 (14.7%)	<0.01
**Etiology for mechanical ventilation**				
Neurological surgery	369 (38.4%)	157 (37.1%)	55 (10.2%)	<0.01
Medical condition	594 (61.7%)	267 (63.0%)	484 (89.8%)	
**Severity scores**				
ICU APACHE II	25.0 ± 6.0	25.7 ± 6.1	24.5 ± 5.8	<0.01
RCC APACHE II	17.8 ± 5.5	19.4 ± 5.7	16.5 ± 5.1	<0.01
**Do-not-resuscitate status**	430 (44.7%)	250 (59.0%)	180 (33.4%)	<0.01
**RCC data (day 1)**				
White blood cell counts (/ml)	1,0881.0 ± 5,001.3	11,279.6 ± 5,307.1	10,567.5 ± 4,728.3	0.03
Hematocrit (%)	29.6 ± 5.2	29.0 ± 5.1	30.1 ± 5.2	<0.01
Creatinine (mg/dl)	1.6 ± 1.8	1.7 ± 1.9	1.4 ± 1.7	<0.01
Sodium (mg/dl)	138.7 ± 6.3	139.1 ± 6.9	138.3 ± 5.8	0.06
Potassium (mg/dl)	4.3 ± 0.7	4.3 ± 0.7	4.3 ± 0.6	0.25
GCS (eye opening)	3.0 ± 1.1	3.0 ± 1.1	3.1 ± 1.0	0.37
GCS (motor response)	4.4 ± 1.7	4.2 ± 1.7	4.6 ± 1.6	<0.01
FiO_2_ (%)	37 ± 5	38 ± 6	36 ± 5	<0.01
Hear rate	87.8 ± 20.5	90.1 ± 20.7	85.9 ± 20.2	<0.01
Respiratory rate	19.1 ± 5.9	19.6 ± 6.1	18.7 ± 5.8	0.01
Blood pressure (systolic)	123.3 ± 23.3	122.4 ± 24.0	124.1 ± 22.7	0.24
Blood pressure (diastolic)	69.0 ± 18.8	67.9 ± 19.1	69.8 ± 18.5	0.12
**Outcome**				
ICU day	23.7 ± 13.1	24.4 ± 15.4	23.1 ± 10.9	0.11
RCC stay	16.7 ± 9.5	19.7 ± 10.7	14.3 ± 7.6	<0.01
Ventilator day	41.7 ± 17.7	50.7 ± 17.9	34.6 ± 14.0	<0.01
Hospital day	52.6 ± 18.0	53.9 ± 18.5	51.6 ± 17.6	0.05
Mortality	180 (18.7%)	164 (38.7%)	16 (3.0%)	<0.01

*Data were presented as mean ± standard deviation and number (percentage)*.

*COPD, chronic obstructive pulmonary disease; ICU, intensive care unit; APACHE II, Acute Physiology and Chronic Health Evaluation II; RCC, respiratory care center; GCS, Glasgow Coma Score; FiO_2_, fraction of inspired oxygen*.

**Table 2:** Weekly ventilatory parameters of the 963 patients categorized by weaning outcome.

	**All**	**Successful weaning (–)**	**Successful weaning (+)**	***p*-value**
	***N* = 963**	***N* = 424**	***N* = 539**	
**FiO_2_ (%)**				
Week 4	35.1 ± 5.6	36.4 ± 6.1	34.1 ± 5.0	<0.01
Week 5	34.9 ± 7.0	36.9 ± 8.8	33.4 ± 4.7	<0.01
Week 6	35.1 ± 8.6	37.6 ± 11.4	33.1 ± 4.8	<0.01
Week 7	35.6 ± 10.3	38.6 ± 13.4	33.2 ± 6.2	<0.01
Week 8	35.8 ± 11.1	39.2 ± 14.5	33.2 ± 6.3	<0.01
Week 9	36.0 ± 12.0	39.7 ± 15.7	33.2 ± 6.8	<0.01
**PEEP (cmH**_**2**_**O)**				
Week 4	5.8 ± 1.6	6.0 ± 1.7	5.6 ± 1.5	<0.01
Week 5	5.6 ± 1.4	5.9 ± 1.6	5.4 ± 1.1	<0.01
Week 6	5.6 ± 1.3	5.9 ± 1.5	5.3 ± 1.0	<0.01
Week 7	5.5 ± 1.3	5.8 ± 1.6	5.2 ± 0.9	<0.01
Week 8	5.5 ± 1.3	5.8 ± 1.6	5.2 ± 0.9	<0.01
Week 9	5.5 ± 1.3	5.9 ± 1.6	5.2 ± 0.9	<0.01
**Ppeak (cmH**_**2**_**O)**				
Week 4	21.7 ± 4.9	23.2 ± 4.9	20.5 ± 4.5	<0.01
Week 5	20.9 ± 5.6	23.2 ± 6.3	19.1 ± 4.1	<0.01
Week 6	20.6 ± 5.4	23.0 ± 5.8	18.6 ± 4.1	<0.01
Week 7	20.6 ± 5.7	23.4 ± 6.3	18.4 ± 4.1	<0.01
Week 8	20.7 ± 5.79	23.7 ± 6.5	18.3 ± 4.0	<0.01
Week 9	20.8 ± 6.0	24.0 ± 6.6	18.3 ± 3.9	<0.01
**Pmean (cmH**_**2**_**O)**				
Week 4	10.6 ± 2.4	11.3 ± 2.5	10.2 ± 2.3	<0.01
Week 5	10.4 ± 2.5	11.3 ± 2.9	9.6 ± 1.9	<0.01
Week 6	10.3 ± 2.6	11.4 ± 3.0	9.4 ± 1.9	<0.01
Week 7	10.3 ± 2.7	11.5 ± 3.2	9.3 ± 1.8	<0.01
Week 8	10.3 ± 2.9	11.6 ± 3.4	9.2 ± 1.8	<0.01
Week 9	10.3 ± 2.9	11.7 ± 3.5	9.2 ± 1.7	<0.01
**VT/PBW (ml/kg)**				
Week 4	9.0 ± 1.9	9.1 ± 1.9	8.9 ± 2.0	0.12
Week 5	8.7 ± 2.0	8.9 ± 2.0	8.5 ± 2.0	<0.01
Week 6	8.6 ± 2.1	8.9 ± 2.0	8.3 ± 2.1	<0.01
Week 7	8.6 ± 2.2	9.0 ± 2.2	8.3 ± 2.1	<0.01
Week 8	8.6 ± 2.2	9.0 ± 2.3	8.3 ± 2.1	<0.01
Week 9	8.6 ± 2.3	9.1 ± 2.4	8.3 ± 2.1	<0.01
**Respiratory rate (/min)**				
Week 4	18.9 ± 3.2	19.0 ± 3.3	18.9 ± 3.2	0.44
Week 5	19.4 ± 3.2	19.3 ± 3.4	19.4 ± 3.1	0.66
Week 6	19.6 ± 3.2	19.4 ± 3.5	19.7 ± 3.0	0.26
Week 7	19.6 ± 3.3	19.5 ± 3.6	19.7 ± 3.1	0.38
Week 8	19.6 ± 3.4	19.4 ± 3.7	19.7 ± 3.1	0.22
Week 9	19.5 ± 3.3	19.2 ± 3.6	19.6 ± 3.0	0.07
**Minute ventilation (L/min)**				
Week 4	9.3 ± 2.5	9.6 ± 2.1	9.1 ± 2.8	<0.01
Week 5	9.2 ± 2.5	9.6 ± 2.4	8.8 ± 2.6	<0.01
Week 6	9.1 ± 2.7	9.6 ± 2.6	8.7 ± 2.7	<0.01
Week 7	9.1 ± 2.9	9.6 ± 2.7	8.6 ± 2.9	<0.01
Week 8	9.0 ± 2.9	9.5 ± 2.9	8.6 ± 2.9	<0.01
Week 9	9.0 ± 3.0	9.6 ± 3.0	8.6 ± 2.9	<0.01

*Data were presented as mean ± standard deviation*.

*FiO_2_, fraction of inspired oxygen; PEEP, positive end-expiratory pressure; Ppeak, peak inspiratory pressure; Pmean, mean airway pressure; VT/PBW, tidal volume per predicted body weight*.

### Comparisons Among XGBoost, RF, and LR

**Figure 1:**
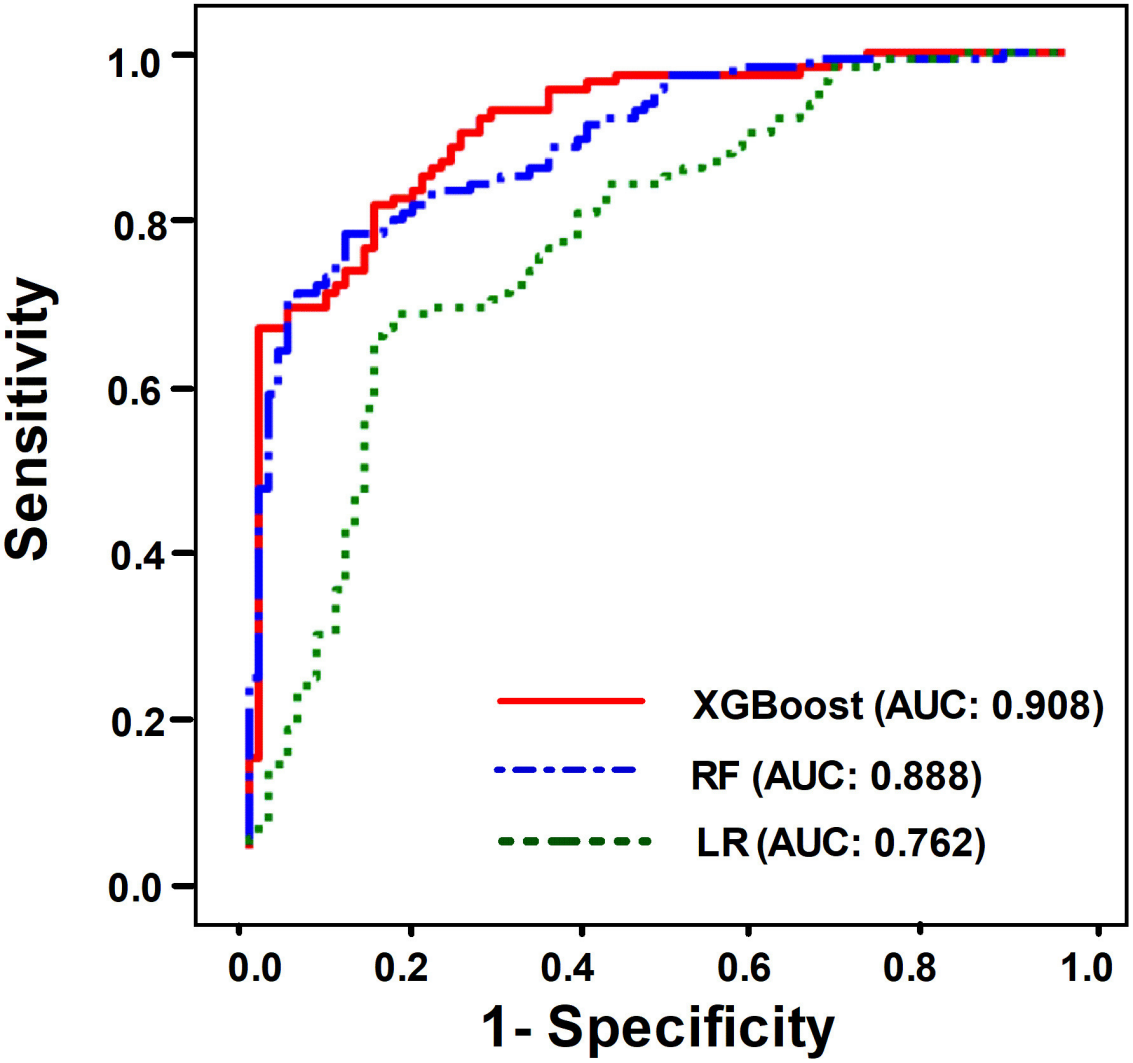
ROC curves demonstrating the performance of the XGBoost model (AUC: 0.908, 95% CI 0.864–0.943), RF (AUC: 0.888, 95% CI 0.844–0.934), and LR (AUC 0.762, 95% CI 0.687–0.830) for predicting successful weaning in patients requiring PMV.

We then compared the performance of the three ML models to predict successful weaning. Using ROC analysis, we found that the AUC value for predicting successful weaning in the XGBoost was 0.908 (95% CI 0.864–0.943), which was similar with the accuracy in RF (AUC: 0.888, 95% CI 0.844–0.934) and better than those in LR (AUC: 0.762; 95% CI 0.687–0.830) (Figure 1) (see Supplementary Table 2 for the detailed metric of the performance). Moreover, we also used DeLong's test to determine the difference between two AUCs and confirmed that XGBoost was similar with RF and outperformed LR (XGBoost against RF, *p* = 0.36; XGBoost against LR, *p* < 0.01).

### Explanation of the Model at the Feature Level

**Figure 2:**
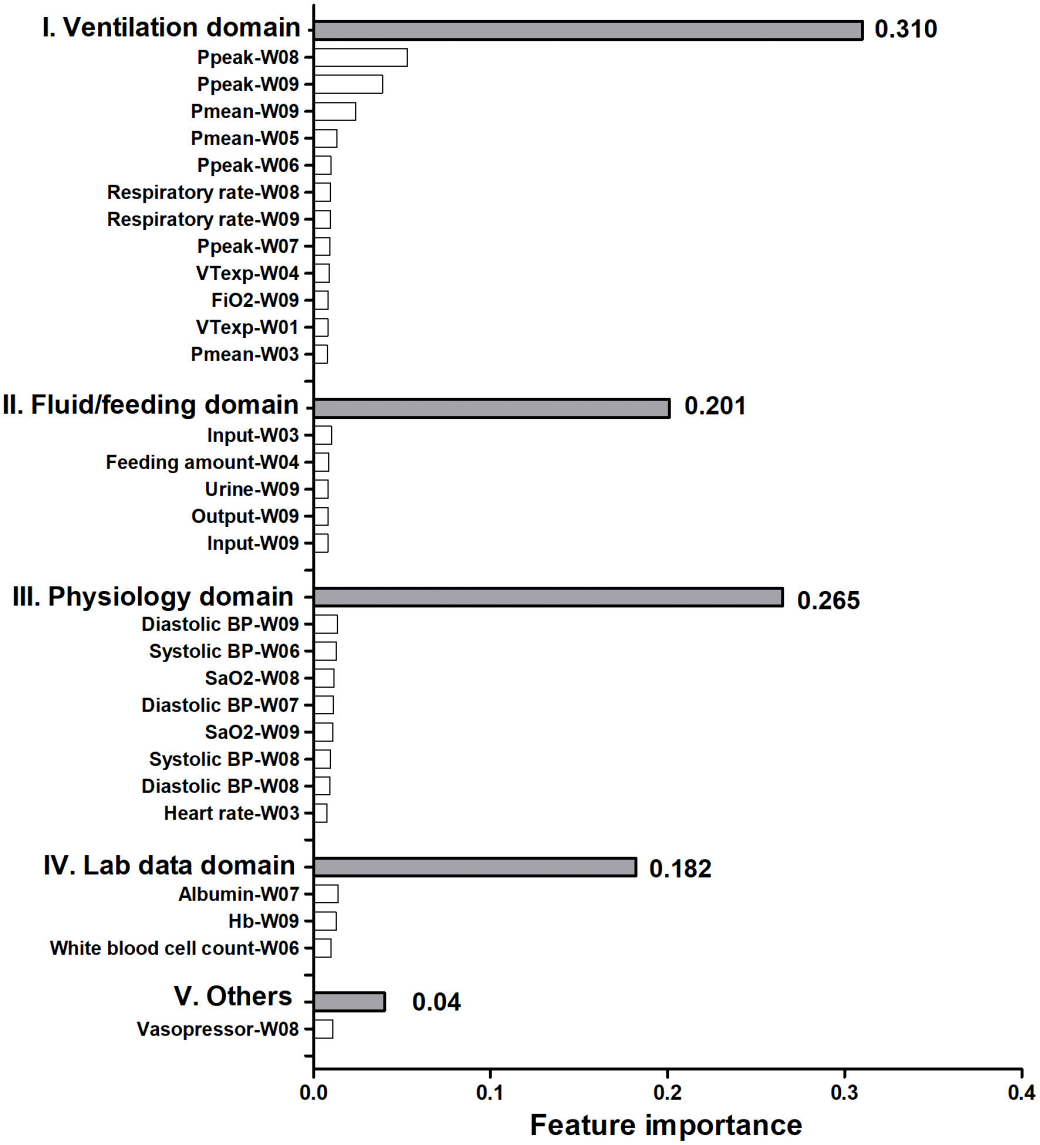
Relative feature importance of the top 30 features categorized by main clinical domains.

**Figure 3:**
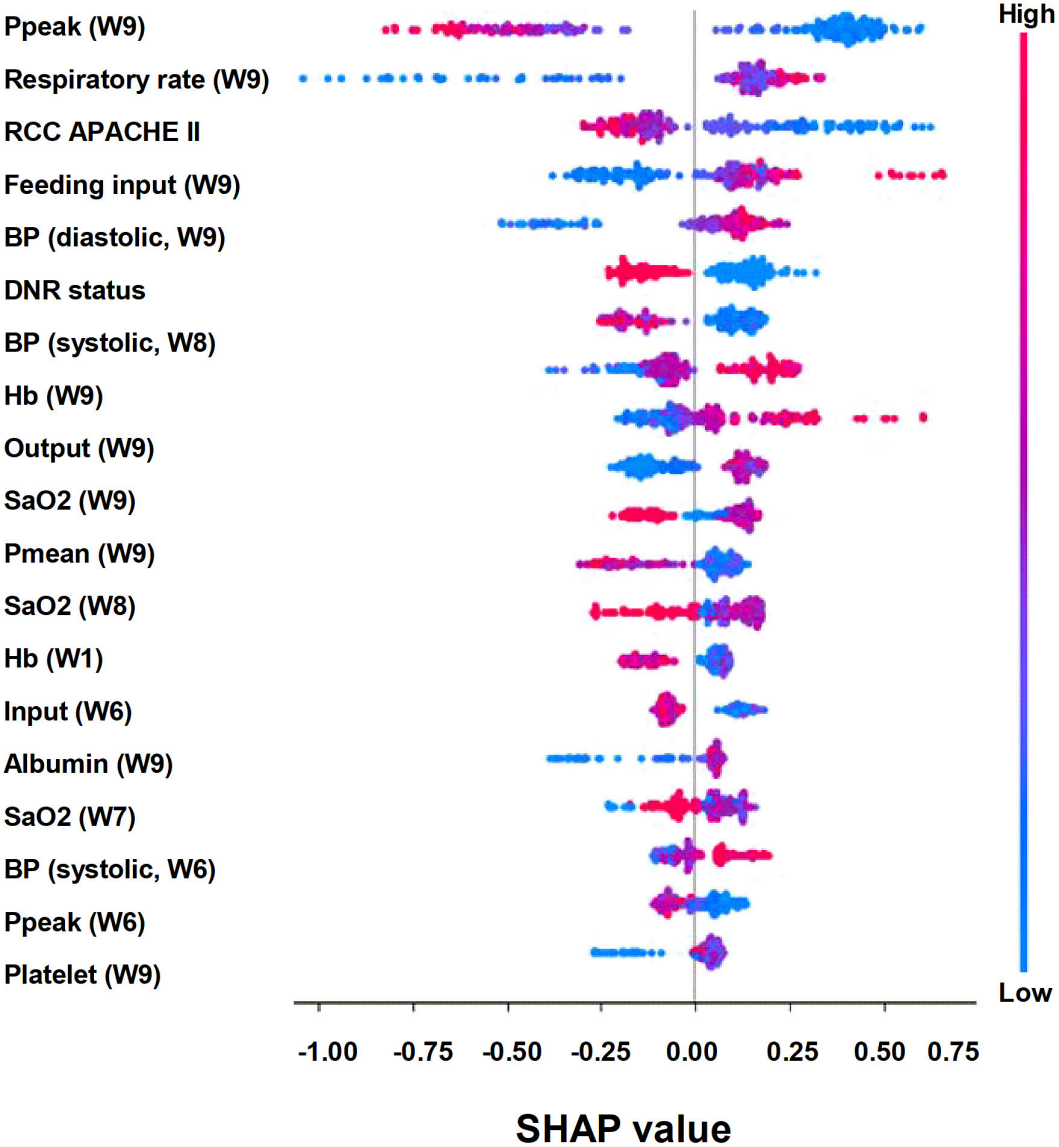
SHAP to illustrate successful weaning prediction model in the feature level.

**Figure 4:**
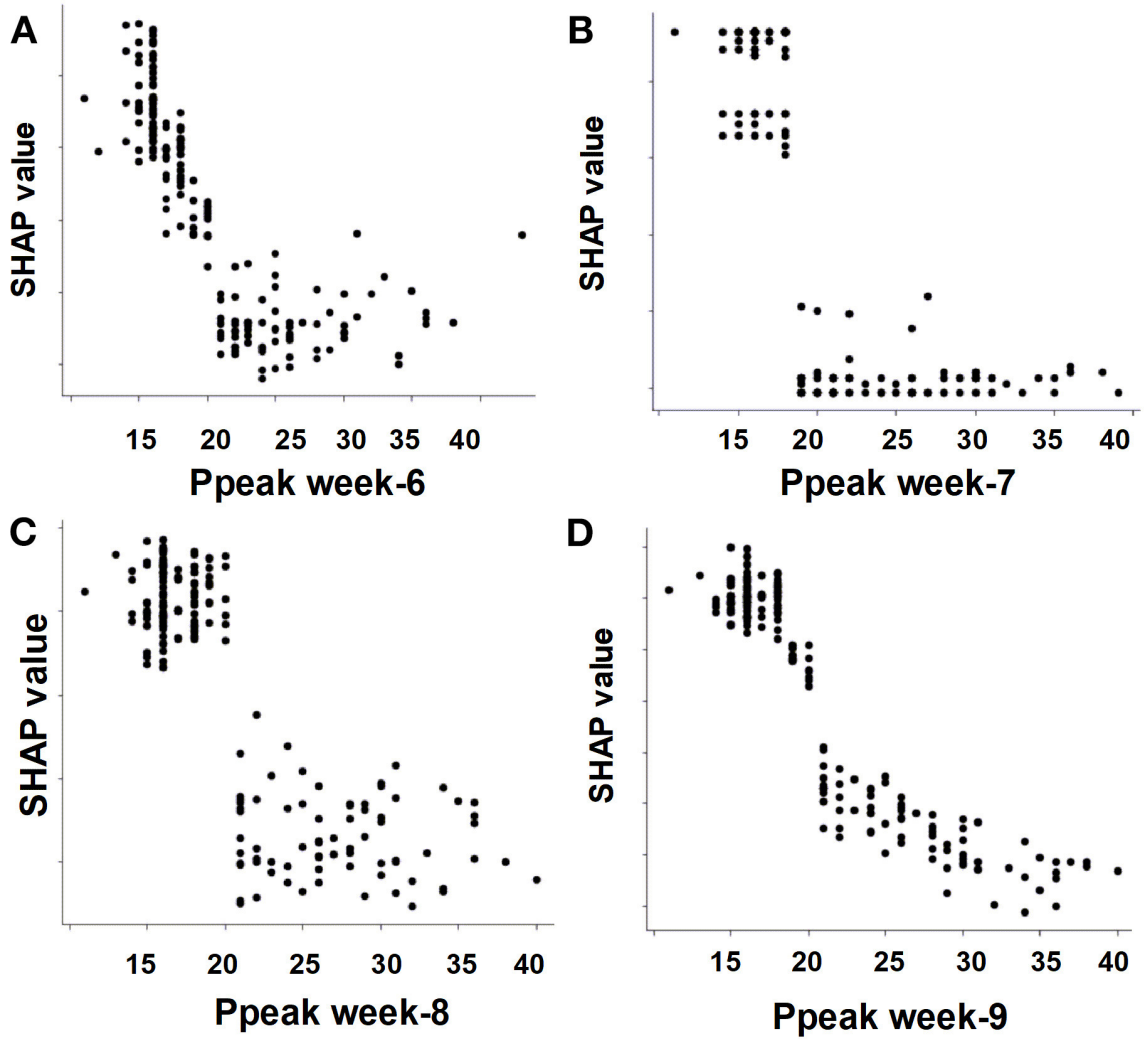
Partial dependence plot by the SHAP value of the weekly Ppeak in predicting successful weaning. **(A)** Week-6, **(B)** week-7, **(C)** week-8, **(D)** week-9.

To give clinicians an intuitive understanding of the established models, we provided a visualized explanation of the model at the clinical domain level, feature level, and individual level. We categorized the top 30 features by main clinical domains (Figure 2). The cumulative feature importance of the ventilatory domain, fluid domain, physiology domain, laboratory data domain, and other domains was 0.310, 0.201, 0.265, 0.182, and 0.04, respectively. Moreover, to enable the visualized interpretation of key features of the model, we used a SHAP plot to illustrate how these features affect weaning outcome (Figure 3). Therefore, the strength and direction of each feature were clearly illustrated in the SHAP plot. For example, a lower Ppeak on week 9 was associated with a higher probability of successful weaning. In addition to using a SHAP plot to demonstrate the direction of the impact of key features, we also used PDP to illustrate how each feature affects the model. As shown in Figure 4, a Ppeak higher than ~20 cmH_2_O was inversely correlated with successful weaning, and such associations were consistent in distinct weeks (Figure 4). Taken together, these visualized interpretations provide explanations of the established model at the clinical domain level and feature level.

### Explanation of the Model at the Individual Level

**Figure 5:**
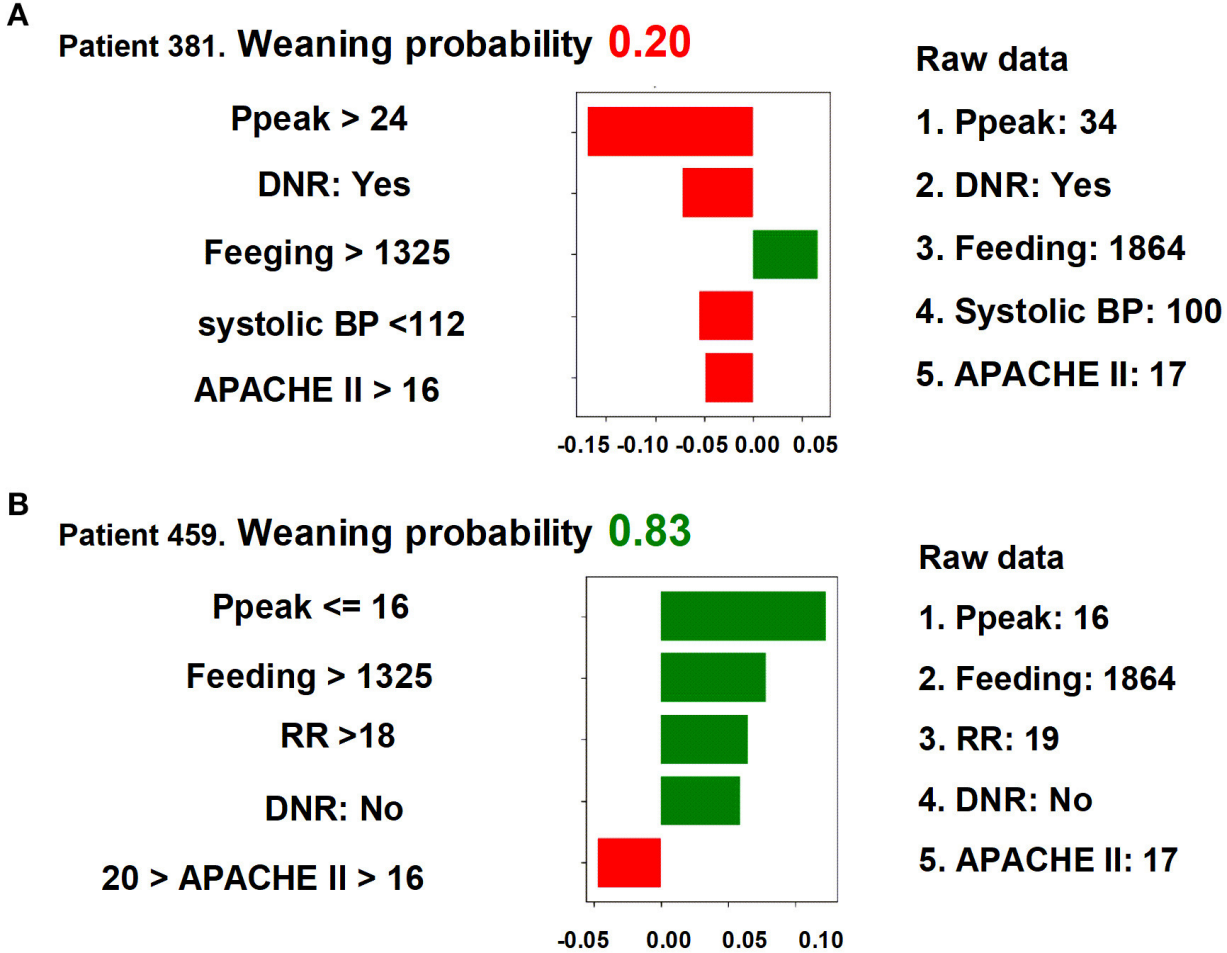
LIME plots of two representative individuals. **(A)** Patient 381, **(B)** patient 459.

We next used LIME to illustrate the impacts of key features on the weaning prediction model in individual patients. As shown in Figure 5, the overall predicted probability of successful weaning (top), true values of the five main features (right), and the classification details (left) of two representative patients were illustrated in the LIME plot. For example, in patient 381, the predicted probability for successful weaning was low (0.20) due to a number of negative conditions, consisting of a high Ppeak (34 cmH_2_O, >24 cmH_2_O), a do-not-resuscitate (DNR) status, a low systolic blood pressure (100 mmHg, <112 mmHg), and a high APACHE II score (17, >16), although there was a good feeding amount (1,864 cm^3^/day, >1,325 cm^3^/day). In contrast, the weaning probability in patient 459 was high (0.83) due to positive conditions, including a low Ppeak (16 cmH_2_O, ≤ 16 cmH_2_O), a high feeding amount (1,864 cm^3^/day, >1,325 cm^3^/day), a high respiratory rate (RR) (19/min, >18/min), and absence of a DNR status, despite a slightly high APACHE II (17, >16). These explanations at the individual level were consistent with the aforementioned explanations at the feature level and should further mitigate the black-box concern.

### Accuracy of the Weekly Weaning Prediction Model

**Figure 6:**
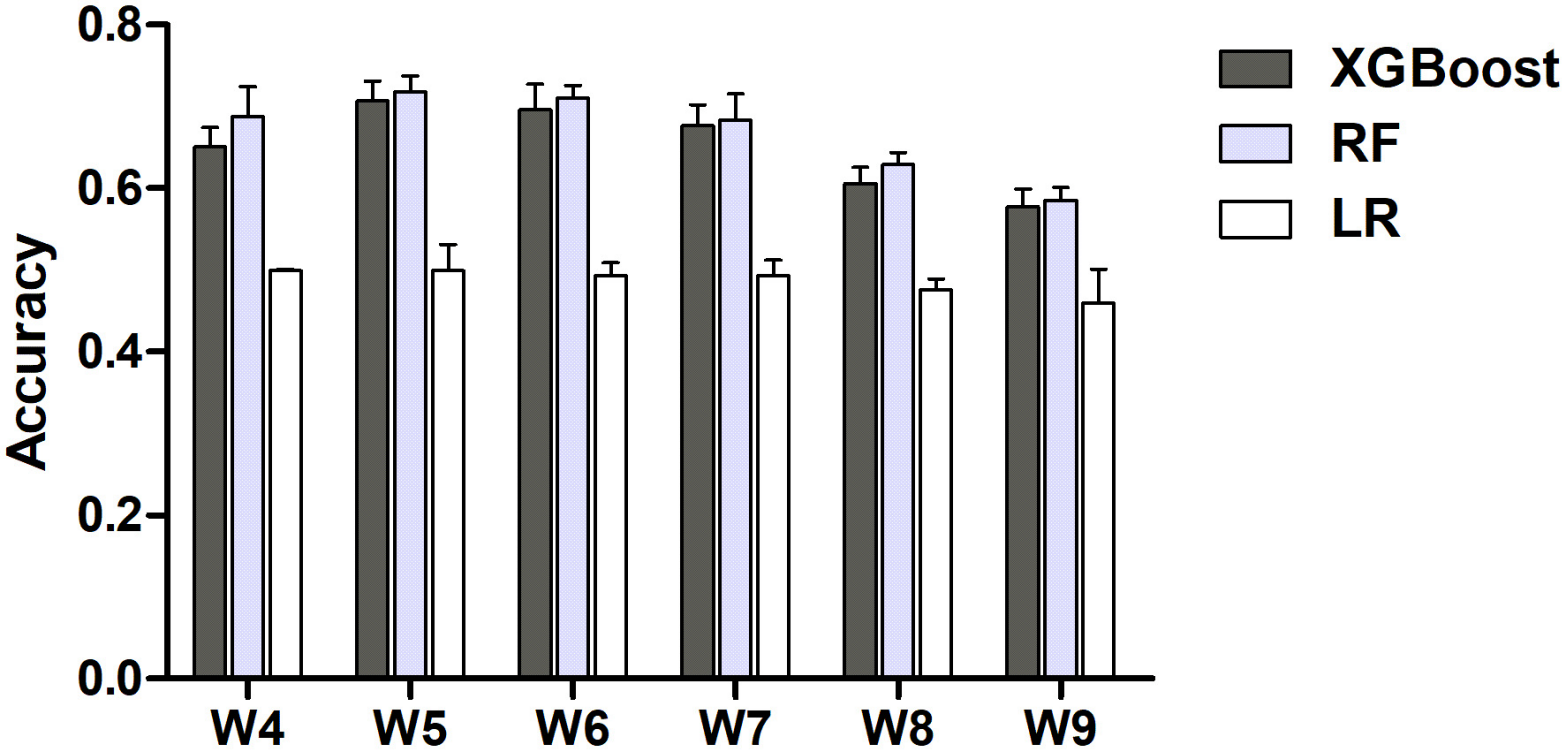
Weekly performance to predict weaning outcome among distinct machine learning models.

To test the performance of real-time prediction with a 7-day prediction window in the proposed weaning outcome prediction model, we analyzed the accuracy of the weekly prediction model (19). In brief, we measured the performance of the three ML models to predict successful weaning on one selected week using data prior to this selected week. In line with the aforementioned findings (Figure 1), the performance was similar between XGBoost and RF, and a lower accuracy was found in the LR model than that in XGBoost/RF (Figure 6). The accuracy of XGBoost and RF was ~0.7 between weeks 4 and 7 and slightly declined to 0.6 on weeks 8 and 9. The domain-based distribution of feature importance and the SHAP plot of the weekly prediction model were also compatible with those in the aforementioned prediction model (Supplementary Figure 2, Figures 3, 4). Collectively, these data demonstrated the feasibility of integrating the proposed ML model into clinical practice in RCC to timely predict the probability of successful weaning.

## Discussion

This study aimed to establish the outcome prediction model in patients requiring PMV through using the explainable ML approach. We found that the accuracy of the XGBoost and RF in predicting successful weaning was high, whereas a relatively low accuracy was found in the LR model. Feature importance analyses illustrated the substantial features based on clinical domains, and SHAP and PDP plots further demonstrated the expected distribution of the impact of each feature in the XGBoost. In addition to the aforementioned interpretability at the feature level, we further used LIME for individual-level interpretability. Furthermore, we addressed the accuracy of the weekly prediction model and found a modest high accuracy to predict successful weaning between weeks 4 and 7. Our findings suggest a practical application of using inherently interpretable ML models to establish a decision support system, particularly in making a high-stake medical decision, given that directly explaining the black-box model remains a niche (20).

Patients requiring PMV is currently a growing issue in Taiwan as well as the world. The advance of critical care has led to not only a steady decrease of the mortality rate among critically ill patients in the past two decades but also an unexpected increase in the number of patients requiring PMV (21, 22). Hill et al., conducting a Canadian population-based cohort study through investigating 213,680 patients who received MV between 2002 and 2013, reported that 5.4% (11,594) of these patients required PMV (23). Furthermore, Damuth et al., conducting a meta-analysis consisting of 39 studies, reported that the pooled proportion of weaning from MV in patients requiring PMV was 50% (6). Lai et al., investigating 27,654 patients receiving MV in southern Taiwan between 2006 and 2014, found that 6.58% (1,821) of them required PMV, and the hospital mortality in those requiring PMV was 17.6% (24). In the present study, the overall weaning rate and hospital mortality rate in patients requiring PMV were 56 and 18.7%, respectively, and these data were consistent with the aforementioned studies in Taiwan as well as the world. These pieces of evidence highlight an increasing burden of patients requiring PMV worldwide and the crucial need to establish the weaning outcome prediction model in patients with PMV.

Indeed, patients with PMV have distinct ventilatory and physiological alternations from those in the acute status of critical illness; therefore, evidence derived from studies conducted in ICUs, focusing on acute resuscitation-relevant characteristics, is unlikely to be extended to those with PMV (25). Notably, unlike the high weaning rate of up to nearly 85% in patients with acute illness (26, 27), the weaning rate in patients requiring PMV was merely 50% (6). Thus, there is an essential need to establish a PMV-specific weaning outcome prediction decision support system (28). Given the distinct physiological characteristics in patients with PMV, a specialized weaning unit, including respiratory intermediate ICUs (RIICUs) and RCC, is required to facilitate weaning in patients with PMV through a team approach, including respiratory therapists, nutritionists, psychologists, and speech and occupational therapists (29). We believe that the established explainable ML model using multidomain real-world data in the specialized weaning unit should be a practical weaning prediction model to facilitate weaning in patients requiring PMV. Weaning success has been defined as consecutive ventilator-free days for 1–7 days in studies regarding weaning. Ruan et al., using a Taiwanese population-based database in one governmental project aiming to investigate MV use in Taiwan, found that the probabilities of the reinstitution of MV for the initial 7 days after ventilator liberation were 25, 8, 3, 3, 2, 1, and 1% in the PMV cohort (12). Therefore, we used liberation from MV for five consecutive days to define a successful weaning success in this study conducted in central Taiwan.

In this study, we identified a similar test accuracy between XGBoost (AUC: 0.908) and RF (AUC: 0.888), whereas the accuracy of LR was relatively low (AUC: 0.762) (Figure 1). The LR model is based on assumptions including the independence between input variables and a linear correlation between input and output variables; therefore, the real-world dataset in medical practice may not meet the assumptions of LR. Instead, tree-based classifiers, including XGBoost and RF, based on homogeneity, should be more likely to meet the characteristics of the dataset in the present study. Given that similar performances were found between RF and XGBoost, we think that the use of regularization, applying the Taylor expansion to approximate the loss function, and high flexibility for fine-tuning might enable XGBoost to perform slightly better than RF.

Although AI technologies have achieved extraordinary advancement in a number of fields, the adoption of AI algorithms with the black-box issue in health care remains uncommon mainly due to physicians tending to take action only after realizing the rationale behind the results (30, 31). Given that an incorrect medical decision can lead to catastrophic effects, particularly in critical care medicine, the black-box aspect somehow leads physicians to distrust the AI model when there is no rationale given behind it (7). Clearly, physicians should reserve their judgements in decision making, and we think the interpreted models, including neural networks, which predict patient outcomes (e.g., patient unlikely to liberate MV due to a high Ppeak and low blood pressure) in accordance with the workflow of physicians' daily practice, should be a crucial supporting element in the overall decision process of physicians (32). Therefore, explainable AI algorithms have been increasingly developed for health care applications, aiming not only to establish a predictive model but also to provide justifications for the prediction in a format that physicians can understand (32, 33). In line with our study, Xie et al. recently proposed a framework of automatic clinical score creation to develop 9–12 variables with the interpretability mortality prediction ML model in critically ill patients through using data of the Medical Information Mart for Intensive Care (MIMIC) III database, a widely used critical care database (34). The aforementioned study conducted by Xie et al. and also our study highlight the use of a reasonable number of features to establish a practical model, given that a high number of features may lead to not only the complexity of the model but also to the difficulty in practical landing (34). Similarly, Roimi et al. recently used 50 key features from 7,000 features in two critical care databases to establish a prediction model for bloodstream infections in critically ill patients (35). Indeed, the black-box issue could not be fully clarified; therefore, the *post-hoc* interpretability should at least mimic the real-world behavior of physicians, rather than merely providing explanations of the logical concepts behind the black box. The LIME method offers an interpretable representation with local fidelity. Notably, LIME is model-agnostic and has been increasingly adopted for interpretable data representation (18). Given that the glass-box model is employed in LIME to approximate the black-box model, the quality of the local fit of the glass-box model to the data could not be controlled and objectively assessed (36).

In addition to weaning, end-of-life care is also a crucial issue in patients requiring PMV, particularly those with difficulty weaning in RCC/RIICU given that prolonged use of ventilator with a low possibility of weaning might lead to medical futility (37). Early integrated palliative care has been found to improve quality of life, to reduce intensive life-sustaining treatments, and to improve caregivers' psychological symptoms (38). We found a declining accuracy in predicting successful weaning in weeks 4–7 (Figure 6); we hence established the mortality prediction model using the same dataset and explainable ML approach. We found that the accuracy to predict mortality was higher than that to predict successful weaning (Supplementary Figure 5). Notably, the high-ranking features to predict mortality appeared to be distinct from that used to predict successful weaning. We found that the DNR status had the highest feature importance in the mortality prediction model, whereas the DNR status was the sixth highest feature importance in the weaning prediction model (Supplementary Figure 6, Figure 3). Indeed, the consensus for DNR is an essential issue among patients requiring PMV, particularly those with a low possibility of weaning. Nava et al., investigating 6,008 patients in European respiratory intermediate care units and high-dependency units, found that merely 21% of patients received end-of-life decision, including withholding of treatment, DNR/do-not-intubate orders, and non-invasive MV (37). Furthermore, studies have shown that timely communication with families and the interprofessional collaboration for individualized balance between aggressiveness and responsiveness of care, which was recently reported by Rak et al. through conducting a large and delicate ethnographic study in eight long-term acute care hospitals, are crucial in the end-of-life care among patients requiring PMV (39, 40). Therefore, we think that the mortality prediction model and the illustration of main features attributed to high mortality in patients with PMV might indicate the need for timely communication regarding end-of-life issues.

There are limitations in this study. First, this study is a single-center study, and external validation is hence needed. However, the overall weaning and mortality rates were similar to those of previous studies, and the used data were routinely collected data in a real-world setting; the concern with regard to generalization should be largely mitigated. Second, some weaning-relevant data, such as rehabilitation programs, were not included in the dataset. We think the accuracy of the model could be further improved after including the aforementioned data; however, the structured data in a real-world setting remain fundamental in the practical landing of the proposed ML mode. Third, the technology readiness level (TRL) of the proposed explainable ML model should merely be TRL-4 (41); however, we believe that the feasibility of practical use with optimal user interface (TRL-5) should be high given that the variables used in this study were obtained from structured electronic medical records of real-world practice at an RCC. Fourth, the number of subjects was relatively small. Given that merely 5–10% of patients receiving MV require PMV, the sample size in studies focusing on PMV is generally small (1, 2). To mitigate the issue of a small sample size, we have performed a grid search for optimal parameters of XGBoost, RF, and LR and have provided metrics of performance in an independent test cohort (80/20 splitting) to show the acceptable accuracy, Brier score, precision, recall, and F1 score in XGBoost/RF (Supplementary Table 2, Supplementary Figure 1). Moreover, the observational nature of this study and the medical decision made by the senior attending physician could potentially introduce a confounding effect. Although the individual decision for weaning was made by the attending physician, the weaning protocol and overall weaning process have been certified by the regular external audit at the RCC in Taiwan. Additionally, patients who were transferred from another hospital may be a concern due to data integrity, but we ascertain the ventilatory data of these patients given that the Taiwanese National Health Insurance, a compulsory population-based insurance in Taiwan, has implemented the nationwide Integrated Prospective Payment (IPP) program on patients with PMV since 2000 (12, 42); therefore, in the present study, we used the registered ventilatory data of these patients in the IPP program although the data might be incomplete.

## Conclusion

In conclusion, using a real-world dataset in patients requiring PMV, we found that XGBoost/RF outperformed LR for predicting weaning outcome in patients requiring PMV. We used domain-based cumulative feature importance, SHAP plots, and PDP plots for visualized interpretations at the feature level and LIME plots to illustrate key determinants at the individual level. We believe these approaches should largely mitigate the black-box issue. Future prospective research is warranted for the landing of the proposed model and to translate the advantages of ML models into clinical outcomes of patients requiring PMV.

## Data Availability Statement

The original contributions presented in the study are included in the article/Supplementary Material, further inquiries can be directed to the corresponding author/s.

## Ethics Statement

The studies involving human participants were reviewed and approved by Institutional Review Board of the Taichung Veterans General Hospital (TCVGH: CE19072A). Written informed consent for participation was not required for this study in accordance with the national legislation and the institutional requirements.

## Author Contributions

M-YL, J-LW, M-CC, C-LW, and W-CC: study concept and design. M-YL, C-CL, P-HL, and W-CC: acquisition of data and analysis and interpretation of data. M-YL and W-CC: drafting the manuscript. All authors: contributed to the article and approved the submitted version.

## Conflict of Interest

The authors declare that the research was conducted in the absence of any commercial or financial relationships that could be construed as a potential conflict of interest.

## Abbreviations

AI: artificial intelligence
APACHE: Acute Physiology and Chronic Health Evaluation
AUC: area under the curve
CI: confidence interval
DNR: do not resuscitate
FiO_2_: inspired oxygen
LIME: local interpretable model-agnostic explanations
LR: logistic regression
ML: machine learning
MV: mechanical ventilation
PDP: partial dependence plot
PEEP: positive end-expiratory pressure
Pmean: mean airway pressure
Ppeak: peak inspiratory pressure
PMV: prolonged mechanical ventilation
RCC: respiratory care center
RF: random forest
RIICU: respiratory intermediate intensive care unit
RR: respiratory rate
ROC: receiver operating characteristic
SaO_2_: oxygen saturation
SHAP: SHapley Additive exPlanations
TCVGH: Taichung Veterans General Hospital
TRL: technology readiness level
VT/PBW: tidal volume per predicted body weight
XGBoost: extreme gradient boosting.
