# Supplementary material for: Explainable Machine Learning to Predict Successful Weaning Among Patients Requiring Prolonged Mechanical Ventilation: A Retrospective Cohort Study in Central Taiwan
Source: Front Med (Lausanne). 2021 Apr 23;8:663739. doi: 10.3389/fmed.2021.663739 (PMC8104124; doi:10.3389/fmed.2021.663739)
Supplement: Supplementary file 1 [file Data_Sheet_1.docx]

**Supplemental table and figures**

| **Supplemental table 1. The applied parameters in the XGBoost, random forest and logistic regression** |
| --- |
| **XGBoost**  'base_score': 0.5; 'booster': 'gbtree'; 'colsample_bylevel': 1; 'colsample_bynode': 1; 'colsample_bytree': 1; 'gamma': 0; 'learning_rate': 0.01; 'max_delta_step': 0; 'max_depth': 3; 'min_child_weight': 5; 'missing': None; 'n_estimators': 770; 'n_jobs': 1; 'nthread': None; 'objective': 'binary:logistic'; 'random_state': 0; 'reg_alpha': 0; 'reg_lambda': 1; 'scale_pos_weight': 421/539; 'seed': None; 'silent': None; 'subsample': 1; 'verbosity': 1; 'eval_metric': 'error'  **Random forest** |
| 'bootstrap': True; 'ccp_alpha': 0.0; 'class_weight': None; 'criterion': 'gini'; 'max_depth': 4; 'max_features': 'auto'; 'max_leaf_nodes': None; 'max_samples': None; 'min_impurity_decrease': 0.0; 'min_impurity_split': None; 'min_samples_leaf': 1; 'min_samples_split': 2; 'min_weight_fraction_leaf': 0.0; 'n_estimators': 100; 'n_jobs': None; 'oob_score': False; 'random_state': None; 'verbose': 0; 'warm_start': False |
| **Logistic regression** |
| 'C': 1.0; 'class_weight': None; 'dual': False; 'fit_intercept': True; 'intercept_scaling': 1; 'l1_ratio': None; 'max_iter': 10000; 'multi_class': 'auto'; 'n_jobs': None; 'penalty': 'l2'; 'random_state': None; 'solver': 'saga'; 'tol': 0.0001; 'verbose': 0; 'warm_start': False. |

Abbreviation: Extreme gradient boosting: XGBoost

**Supplemental table 2. Metrics of performance in XGBoost, RF and LR**

|  | Accuracy^a^ | Brier-Score | Precision | Recall | F1-Score |
| --- | --- | --- | --- | --- | --- |
| **XGBoost** | 0.85 | 0.13 | 0.81 | 0.75 | 0.78 |
| **RF** | 0.80 | 0.15 | 0.77 | 0.88 | 0.82 |
| LR | 0.73 | 0.18 | 0.71 | 0.80 | 0.75 |

^a^ (TP+TN) / (TP+FN+TN+FP). Abbreviation: Extreme gradient boosting, XGBoost; random forest, RF; logistical regression, LR.

**Deal with missing values**

1. **Data pre-processing**

**Data of all features were used**

**80/20 splitting**

1. **Training/Test dataset splitting**

**Training set**

**Testing set**

**Grid-search for parameters**

1. **Model training**
2. **XGBoost model**
3. **Random Forest model**
4. **Logistical regression model**

**Prediction model establishment**

**Weaing/Mortality prediction**

**0 (No)/1 (Yest)**

1. **Model prediction**

**Precision/Recall/F1/Brier/AUC computation**

**Supplemental Figure1. Flow diagram of the study**

**Supplemental Figure 2. Relative feature importance categorised by the (five) main clinical domains in model to predict week-5 successful weaning using data prior to week-4.**

**Supplemental Figure 3. SHAP plot of key features used to predict week-5 successful weaning using data prior to week-4.**

**Supplemental Figure 4. Receiver operating characteristic curves demonstrating the performance of the XGBoost model (AUC 0.969, 95% CI 0.946–0.989), RF (AUC 0.937, 95% CI 0.906–0.982), and LR (AUC 0.849, 95% CI 0.765–0.919) to predict mortality in patients requiring prolonged mechanical ventilation.**

**Supplemental Figure 5. SHAP to illustrate mortality prediction model in feature level.**
